# Supplementary figures and images for: Behavioral interplay between mosquito and mycolactone produced by Mycobacterium ulcerans and bacterial gene expression induced by mosquito proximity
Source: PLoS One. 2023 Aug 3;18(8):e0289768. doi: 10.1371/journal.pone.0289768 (PMC10399876; doi:10.1371/journal.pone.0289768)

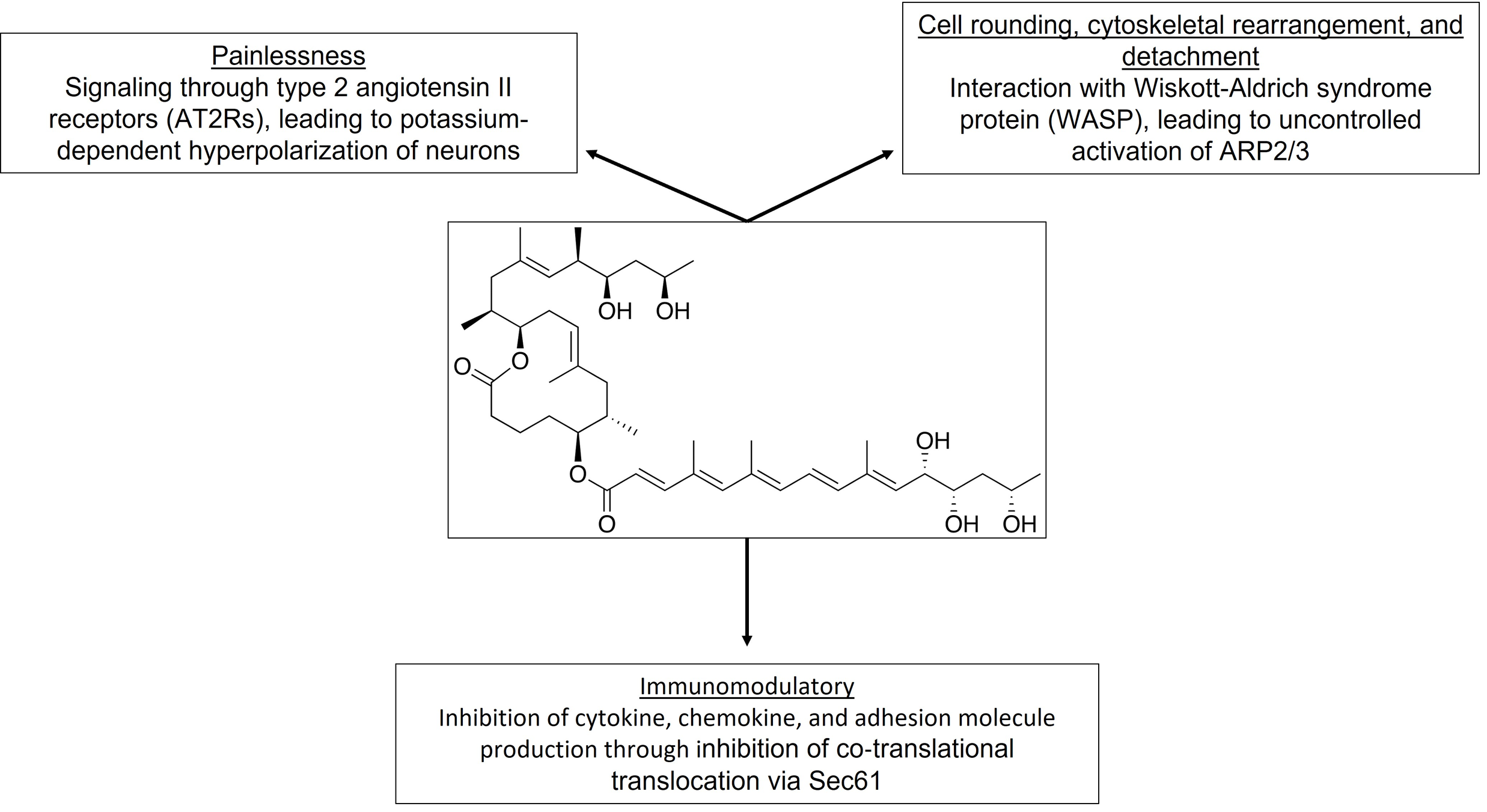

Supplement: S1 Fig — Original mycolactone figure obtained by Lrandolp—Own work, CC BY-SA 3.0, https://commons.wikimedia.org/w/index.php?curid=7007015. (TIF) [file pone.0289768.s001.tif]

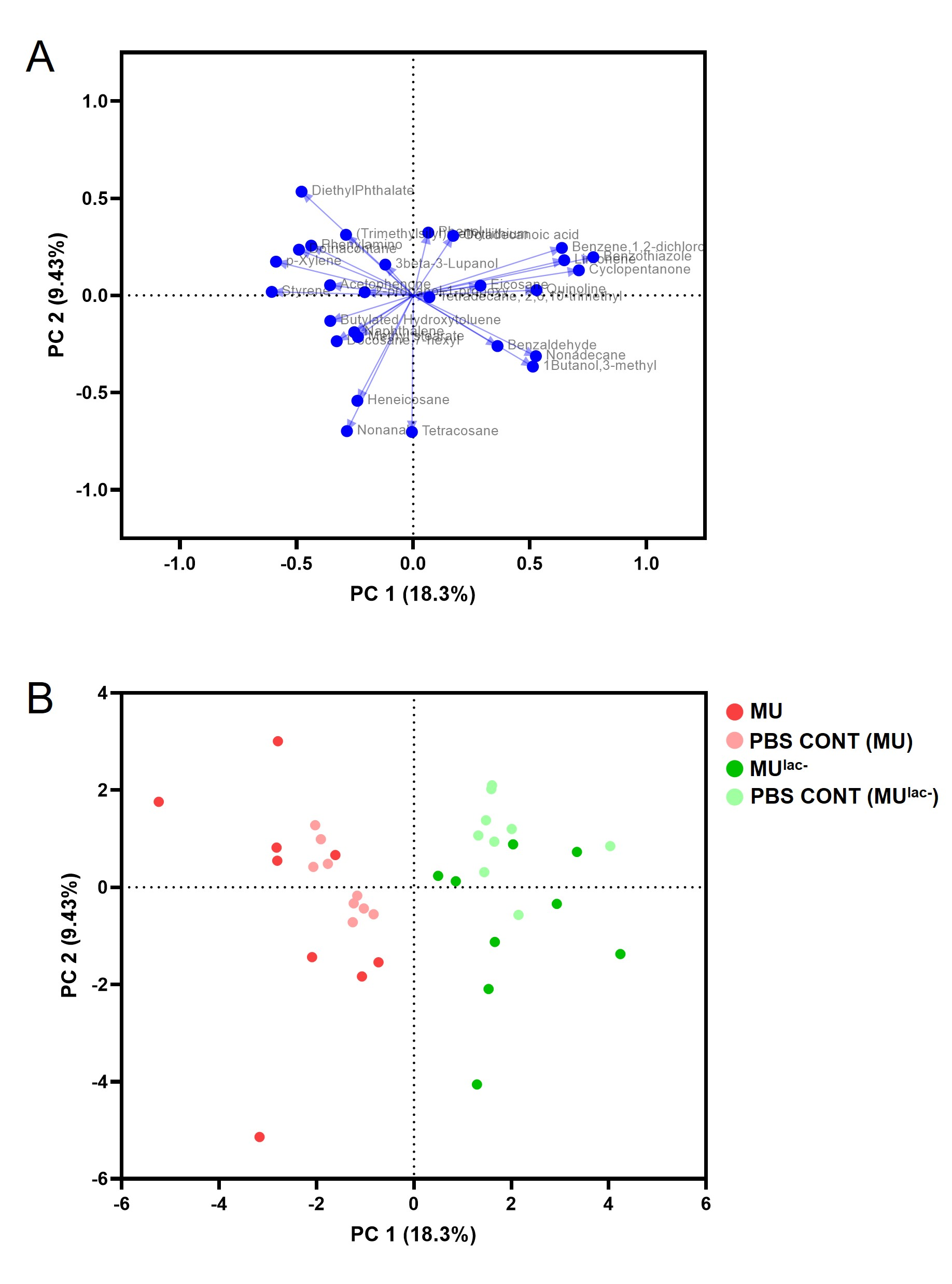

Supplement: S2 Fig — (A) Loading plot displays the relationships and (B) score plot shows the distribution of the samples based on their scores in PC1 and PC2. Replicate samples are represented by dots of the same color. (TIF) [file pone.0289768.s002.tif]

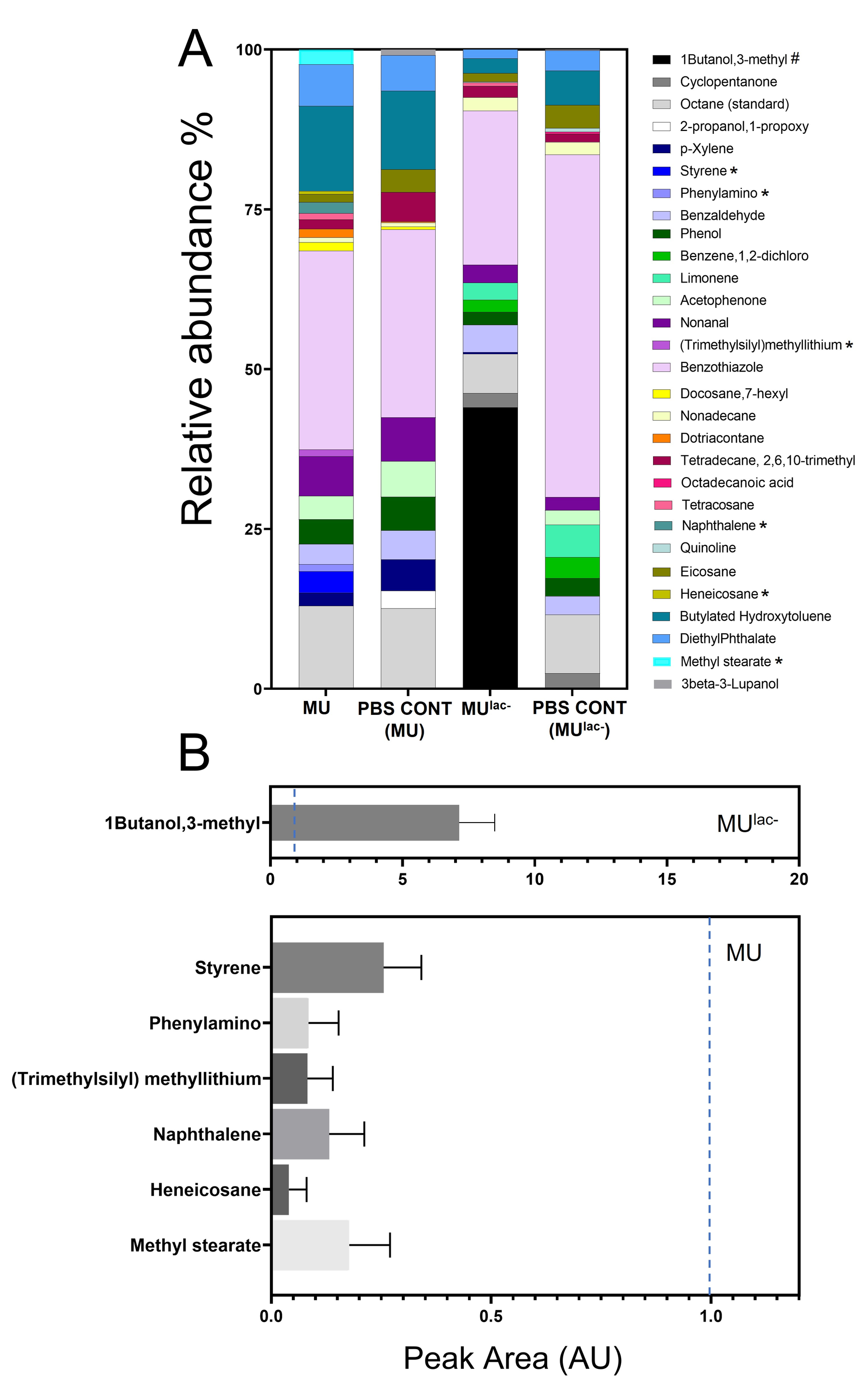

Supplement: S3 Fig — Asterisk (*) and pound sign (#) represent VOCs uniquely expressed by MU or MUlac-, respectively. (B) Bar chart of internal standard ratioed peak area values (Mean ± SEM) for seven selected VOCs that were unique to MU or MUlac- samples. The octane as an internal standard (blue dashed line) is assigned 1 and the others assigned a fractional percent of that value. (TIF) [file pone.0289768.s003.tif]
