# Supplementary material for: Behavioral interplay between mosquito and mycolactone produced by Mycobacterium ulcerans and bacterial gene expression induced by mosquito proximity
Source: PLoS One. 2023 Aug 3;18(8):e0289768. doi: 10.1371/journal.pone.0289768 (PMC10399876; doi:10.1371/journal.pone.0289768)
Supplement: Attachment — Submitted filename: PLOS one.docx [file pone.0289768.s004.docx]

MS entitled, ‘Behavioral interplay between mosquito and mycolactone produced by Mycobacterium ulcerans and bacterial gene expression induced by mosquito proximity’ by Dongmin Kim is really interesting piece for me. I find it very enjoying while reviewing this manuscript. Below are my few queries to be addressed by authors:

1. In introduction, I suggest authors to add more about the mycolactone. Is there any chemical structure available? If yes, please add it. Further, please add its biosynthetic pathway by which it could be produced.
2. Moreover, authors need to add the schematic diagram explaining plausible molecular targets.
3. ‘ ml’ should be written as ‘mL’ throughout the manuscript.
4. Figure 1(B) needs to be replaced with more additional clarified image.
5. Authors can supplement GC-MS results as a supporting material. Or provide for review.
