# Supplementary material for: Behavioral interplay between mosquito and mycolactone produced by Mycobacterium ulcerans and bacterial gene expression induced by mosquito proximity
Source: PLoS One. 2023 Aug 3;18(8):e0289768. doi: 10.1371/journal.pone.0289768 (PMC10399876; doi:10.1371/journal.pone.0289768)
Supplement: Attachment — Submitted filename: Response to Reviewers.docx [file pone.0289768.s005.docx]

**PONE-D-23-13191**

**Behavioral interplay between mosquito and mycolactone produced by Mycobacterium ulcerans and bacterial gene expression induced by mosquito proximity**

**PLOS ONE**

Dear Dr. Kim,

Thank you for submitting your manuscript to PLOS ONE. After careful consideration, we feel that it has merit but does not fully meet PLOS ONE’s publication criteria as it currently stands. Therefore, we invite you to submit a revised version of the manuscript that addresses the points raised during the review process.

Please submit your revised manuscript by Jul 28 2023 11:59PM. If you will need more time than this to complete your revisions, please reply to this message or contact the journal office at plosone@plos.org. When you're ready to submit your revision, log on to https://www.editorialmanager.com/pone/ and select the 'Submissions Needing Revision' folder to locate your manuscript file.

Please include the following items when submitting your revised manuscript:

- A rebuttal letter that responds to each point raised by the academic editor and reviewer(s). You should upload this letter as a separate file labeled '**Response to Reviewers**'.
- A marked-up copy of your manuscript that highlights changes made to the original version. You should upload this as a separate file labeled '**Revised Manuscript with Track Changes'**.
- An unmarked version of your revised paper without tracked changes. You should upload this as a separate file labeled '**Manuscript**'.

If you would like to make changes to your financial disclosure, please include your updated statement in your cover letter. Guidelines for resubmitting your figure files are available below the reviewer comments at the end of this letter.

If applicable, we recommend that you deposit your laboratory protocols in protocols.io to enhance the reproducibility of your results. Protocols.io assigns your protocol its own identifier (DOI) so that it can be cited independently in the future. For instructions see: <https://journals.plos.org/plosone/s/submission-guidelines#loc-laboratory-protocols>. Additionally, PLOS ONE offers an option for publishing peer-reviewed Lab Protocol articles, which describe protocols hosted on protocols.io. Read more information on sharing protocols at <https://plos.org/protocols?utm_medium=editorial-email&utm_source=authorletters&utm_campaign=protocols>.

We look forward to receiving your revised manuscript.

Kind regards,

Mozaniel Santana de Oliveira, Ph.D

Academic Editor

PLOS ONE

**Journal Requirements:**

When submitting your revision, we need you to address these additional requirements.

1. Please ensure that your manuscript meets PLOS ONE's style requirements, including those for file naming. The PLOS ONE style templates can be found at

https://journals.plos.org/plosone/s/file?id=wjVg/PLOSOne_formatting_sample_main_body.pdf and 
<https://journals.plos.org/plosone/s/file?id=ba62/PLOSOne_formatting_sample_title_authors_affiliations.pdf>

2. We note that the grant information you provided in the ‘Funding Information’ and ‘Financial Disclosure’ sections do not match.

When you resubmit, please ensure that you provide the correct grant numbers for the awards you received for your study in the ‘Funding Information’ section.

**We revised the grant information in the manuscript to match financial Disclosure sections.**

3. Thank you for stating the following in your Competing Interests section:  
"NO authors have competing interests"

**Please add this sentence to Competing interests. It is required by the USDA so please add it somewhere: there is no commercial or financial conflict of interest. Mention of trade names, companies, or commercial products in this publication is solely to provide specific scientific information and does not imply recommendation or endorsement of the products by the U.S. Department of Agriculture.**

Please complete your Competing Interests on the online submission form to state any Competing Interests. If you have no competing interests, please state "The authors have declared that no competing interests exist.", as detailed online in our guide for authors at http://journals.plos.org/plosone/s/submit-now 
 This information should be included in your cover letter; we will change the online submission form on your behalf.

4. In your Data Availability statement, you have not specified where the minimal data set underlying the results described in your manuscript can be found. PLOS defines a study's minimal data set as the underlying data used to reach the conclusions drawn in the manuscript and any additional data required to replicate the reported study findings in their entirety. All PLOS journals require that the minimal data set be made fully available. For more information about our data policy, please see http://journals.plos.org/plosone/s/data-availability.

"Upon re-submitting your revised manuscript, please upload your study’s minimal underlying data set as either Supporting Information files or to a stable, public repository and include the relevant URLs, DOIs, or accession numbers within your revised cover letter. For a list of acceptable repositories, please see http://journals.plos.org/plosone/s/data-availability#loc-recommended-repositories. Any potentially identifying patient information must be fully anonymized.

Important: If there are ethical or legal restrictions to sharing your data publicly, please explain these restrictions in detail. Please see our guidelines for more information on what we consider unacceptable restrictions to publicly sharing data: http://journals.plos.org/plosone/s/data-availability#loc-unacceptable-data-access-restrictions. Note that it is not acceptable for the authors to be the sole named individuals responsible for ensuring data access.

We will update your Data Availability statement to reflect the information you provide in your cover letter.

**Data Availability statement has been revised and authors have uploaded the RNA Seq Data openly available in NIH (Bioproject: PRJNA729466) at** [**https://www.ncbi.nlm**](https://www.ncbi.nlm)**. nih.gov/bioproject/PRJNA729466.**

5. We note that you have included the phrase “data not shown” in your manuscript. Unfortunately, this does not meet our data sharing requirements. PLOS does not permit references to inaccessible data. We require that authors provide all relevant data within the paper, Supporting Information files, or in an acceptable, public repository. Please add a citation to support this phrase or upload the data that corresponds with these findings to a stable repository (such as Figshare or Dryad) and provide and URLs, DOIs, or accession numbers that may be used to access these data. Or, if the data are not a core part of the research being presented in your study, we ask that you remove the phrase that refers to these data.

**We added a citation to support this statement (Line # 320).**

6. Please review your reference list to ensure that it is complete and correct. If you have cited papers that have been retracted, please include the rationale for doing so in the manuscript text, or remove these references and replace them with relevant current references. Any changes to the reference list should be mentioned in the rebuttal letter that accompanies your revised manuscript. If you need to cite a retracted article, indicate the article’s retracted status in the References list and also include a citation and full reference for the retraction notice.

**We have thoroughly reviewed all the references and made the necessary revisions in accordance with the PLOS ONE policy.**

[Note: HTML markup is below. Please do not edit.]

Reviewers' comments:

Reviewer's Responses to Questions

**Comments to the Author**

**Dear Reviewers**

**Thank you for giving me the opportunity to submit a revised draft of my manuscript titled " Behavioral interplay between mosquito and mycolactone produced by *Mycobacterium ulcerans* and bacterial gene expression induced by mosquito proximity" to PLOS ONE. We appreciate the time and effort that the reviewers have dedicated to providing valuable feedback on our manuscript. We are grateful to the reviewers for their insightful and thoughtful comments on our paper. We have been able to incorporate changes to reflect most of the suggestions demonstrated by the reviewers. We indicated them by using tracked changes within the manuscript. Here is a point-by-point response to the reviewers’ comments and concerns.**

Review Comments to the Author

Reviewer #1:

MS entitled, ‘Behavioral interplay between mosquito and mycolactone produced by Mycobacterium ulcerans and bacterial gene expression induced by mosquito proximity’ by Dongmin Kim is really interesting piece for me. I find it very enjoying while reviewing this manuscript. Below are my few queries to be addressed by authors:
**The authors greatly appreciate the time and effort taken by the reviewer to improve our submission. Our responses are documented below:**

1. In introduction, I suggest authors to add more about the mycolactone. Is there any chemical structure available? If yes, please add it. Further, please add its biosynthetic pathway by which it could be produced.
**The authors agree and appreciate the reviewer’s insight. The mycolactone descriptions including chemical structure and biosynthesis pathway have been added to introduction (Line # 58).**

2. Moreover, authors need to add the schematic diagram explaining plausible molecular targets.
**Descriptions for the molecular targets have been incorporated into the introduction, specifically on Line 61. Additionally, we have included a schematic diagram related to this information, which has been uploaded as supporting information (S1 Fig).**

3. ‘ml’ should be written as ‘mL’ throughout the manuscript.

**The authors apologize for missing these obvious mistakes. Corrections have been made to the manuscript.**

4. Figure 1(B) needs to be replaced with more additional clarified image.
**The high-resolution image in Figure 1(B) has been updated.**

5. Authors can supplement GC-MS results as a supporting material. Or provide for review.

**GC-MS results including loading/ score plots, Composition ratio, and bar chart of internal standard ratioed peak area values have been uploaded as supporting information.**

Reviewer #2:

The manuscript entitled "Behavioral interplay between mosquito and mycolactone produced by Mycobacterium ulcerans and bacterial gene expression induced by mosquito proximity" is well organized and brings important advances in its field of research. However, some improvements need to be made to make it suitable for publication in this journal.

**The authors thank Reviewer #2 for your thoughtful criticism of our work. In response to these suggestions, we have made incorporated edits when possible and to improve clarity.**

1) The experimental section is very complex, with many details that make the reading tiresome and confusing. I suggest that the author be more succinct in his writing, simplifying and making the text more attractive.

**The authors agree and the experimental section has been revised.**

2) The presence of diethyl phthalate (Table 1) looks like plastic contamination because since the compound is synthetic, it is unlikely to appear naturally in the sample studied. If the authors have used any material used in the extraction of the volatiles made of plastic, I suggest that the identification of the compounds be reviewed.

**You have raised an important point here. The authors concur that diethyl phthalate, which has low volatility, is unlikely to have originated from the samples. It raises suspicion that this compound could have been collected from the parafilm used to seal the glass filtering jar during VOC collection. Table 1 provides evidence that nine compounds, including diethyl phthalate, were present in all treatments.**

3) The data provided in the manuscript are insufficient to determine the identity of the compounds. I suggest that the authors insert chromatograms and the mass spectra (for the majority compounds). This will facilitate the verification of the identity of the chemical compounds.

**We completely agree with your comment. GC-MS results including loading/ score plots, Composition ratio, and bar chart of internal standard ratioed peak area values have been uploaded as supporting materials.**

**Finally, we look forward to responding to any further questions and comments you may have. We thank the reviewers for their time and effort again.**
